# From cortisol-producing adrenal adenoma to atrial myxoma, through nivolumab-induced hypophysitis: a complicated case report of Carney Complex

**DOI:** 10.1007/s12020-024-03997-9

**Published:** 2024-09-01

**Authors:** Ludovico Di Gioia, Giovanni Dambrosio, Angelo Cignarelli, Annalisa Natalicchio, Sebastio Perrini, Luigi Laviola, Francesco Giorgino, Gian Pio Sorice

**Affiliations:** Department of Precision and Regenerative Medicine and Ionian Area, Section of Internal Medicine, Endocrinology, Andrology and Metabolic Diseases, University of Bari Aldo Moro, Bari, Italy

**Keywords:** Carney Complex, hypophysitis, nivolumab, atrial myxoma

## Abstract

**Purpose:**

Carney complex (CNC) is a rare, autosomal dominant syndrome, most commonly caused by *PRKAR1A* gene mutations and characterized by pigmented skin and mucosal changes with multiple endocrine and non-endocrine tumours. This case report highlights the diagnostic challenges associated with CNC in a patient with multiple neoplasms and a complex medical history, including cortisol-producing adrenal adenoma, breast cancer, melanoma, and atrial myxoma.

**Methods:**

We report the case of a 41-year-old woman with a medical history of left adrenalectomy for cortisol producing adenoma (2005) with no sign of adrenal insufficiency at follow-up, right mastectomy for *BRCA1/2* negative carcinoma (2013) and left parotid BRAF-V600E wild-type melanoma (2019), treated with nivolumab adjuvant therapy. In August 2019, following the fifth nivolumab administration, the patient developed central hypocortisolism due to iatrogenic hypophysitis, confirmed by brain MRI and properly treated with oral hydrocortisone. Nivolumab was discontinued due to the patient’s decision. In October 2020 and April 2021, the patient had ischaemic strokes, requiring systemic thrombolysis. Echocardiographic examination then revealed a left atrial mass, with histological finding of myxoma.

**Results:**

Given the rarity of this neoplasm and the suspicion of a syndromic disorder, a genetic evaluation was conducted, which confirmed a *PRKAR1A* gene mutation and the diagnosis of Carney complex.

**Conclusion:**

This case illustrates the diagnostic challenges in CNC, especially in patients with multiple tumourous manifestations and a wide spectrum of life-threatening clinical presentations. It underscores the importance of a multidisciplinary approach to diagnose and manage rare diseases, improving patient outcomes through timely genetic testing and coordinated care.

## Introduction

Carney complex (CNC) is a rare, autosomal dominant syndrome, most commonly caused by heterozygous inactivating mutations of the *PRKAR1A* gene (encoding a key component of the cAMP signalling pathway) on chromosome 17 (17q23-q24), which may function as a tumour-suppressor gene [1].

The clinical manifestations of CNC include pigmented skin and mucosal changes with multiple endocrine and non-endocrine tumours, including primary pigmented nodular adrenocortical disease (a rare cause of Cushing’s syndrome), breast cancer, and atrial myxoma.

Herein, we report a case of CNC with multiple skin tumours, breast cancer and Cushing’s syndrome, which was then complicated by iatrogenic hypophysitis and ischaemic strokes due to atrial myxoma.

## Case report

A 41-year-old woman with previous cortisol-producing adrenal adenoma was initially referred to our clinic for hypophysitis occurring after treatment with nivolumab.

Written informed consent was obtained from the patient for the publication of any potentially identifiable images or data included in this article.

**Fig. 1:**
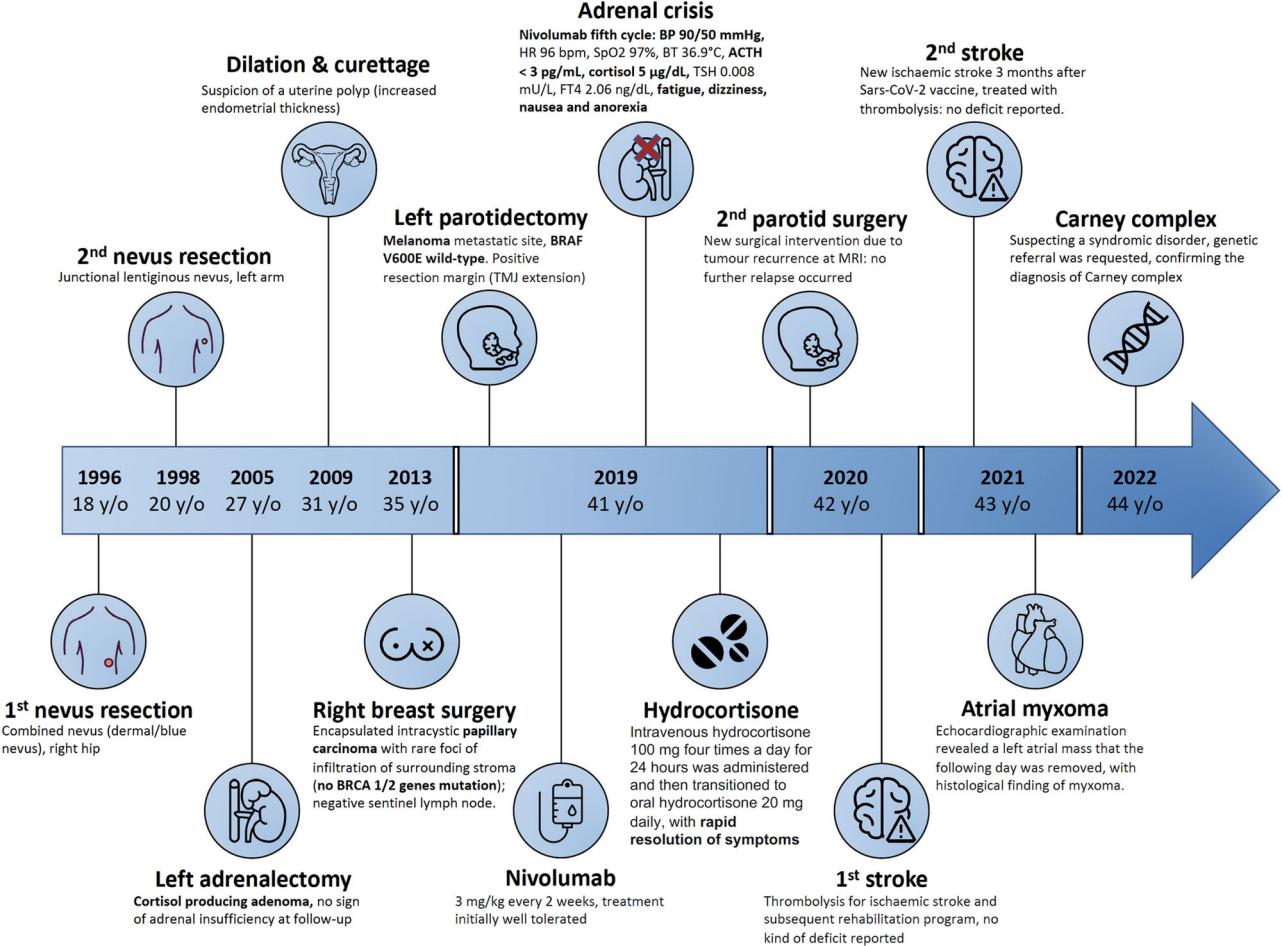
Medical history. BRCA breast cancer, BP blood pressure, HR heart rate, SpO_2_ oxygen saturation, ACTH adrenocorticotropic hormone, TSH thyroid-stimulating hormone, FT4 free thyroxine, MRI magnetic resonance imaging

Her family history included hypertension, myocardial infarction, and breast cancer (mother, at 70 yrs old). Her medical history (Fig. 1) began when she was 18 years old, at which time she had a combined naevus (dermal/blue naevus) removed from her right hip, and then, at age 20, a junctional lentiginous naevus removed from her left arm. The patient had no history of skin myxomas.

She underwent left adrenalectomy in 2005 for an adenoma with a maximum diameter of 23 mm at CT scan, with evidence of basal cortisol hypersecretion and abnormal cortisol response to 1 mg-dexamethasone suppression test on preoperative screening. Follow-up investigations did not reveal any signs of adrenal insufficiency after surgery. No evidence of primary pigmented nodular adrenal dysplasia (PPNAD) was reported in histological examination.

In November 2009, the patient underwent uterine dilation and curettage in suspicion of a uterine cancer; increased endometrial thickness during the follicular phase was reported at that time.

In January 2013, a total breast retro-areolar duct resection with inferior external quadrantectomy was performed, with histological finding of encapsulated intracystic papillary carcinoma with rare foci of infiltration into the surrounding stroma. Therefore, in March 2013, the patient underwent nipple-areola complex sparing mastectomy, with resection margins and sentinel lymph node free from neoplasm (no *BRCA1/2* mutation was found).

In March 2019, following swelling of the left parotid region and discovery of a 30-mm suspicious lesion at CT scan, the patient underwent excision of left parotid with histological diagnosis of BRAF V600E wild-type melanoma with positive resection margins and extension to the temporomandibular joint. In June 2019, treatment with nivolumab (3 mg/kg every 2 weeks) was initiated. Treatment was initially well-tolerated, with no alterations in thyroid function. However, in August 2019, following the fifth nivolumab administration, the patient reported low blood pressure, a high heart rate, extreme fatigue, dizziness, nausea, and anorexia, without headache or visual field defects. Biochemical findings confirmed central hypocortisolism: ACTH < 3 pg/mL, cortisol 5 μg/dL. The patient received intravenous hydrocortisone 100 mg four times a day for 24 hours and then transitioned to oral hydrocortisone 20 mg daily, with a rapid resolution of symptoms. Furthermore, thyroid function tests (Table 1) revealed transient thyrotoxicosis (TSH 0.008 mU/L), FT4 20.6 pg/mL, FT3 3.67 pg/mL, negative anti-thyroid peroxidase, anti-thyroglobulin and anti-TSH receptor antibodies, ultimately evolving into euthyroidism (TSH 1.86 mU/L, FT4 12.04 pg/mL in December 2019) not requiring levothyroxine replacement therapy. There was no evidence of diabetes insipidus.

**Table 1:** Autoimmunity and endocrine assessment

Autoimmunity and endocrine assessments				
	8/2019^a^	12/2019^b^		
Anti-GAD Ab	–	<0.1	UA/mL	0.01–1.01
Anti-TG Ab	<5	<5	UI/mL	5–100
Anti-TPO Ab	<1	<1	UI/mL	1–16
Anti-TSHr Ab	<0.3	–	UI/L	0–1
FT3	3.67	3.39	pg/mL	2.2–4.2
FT4	**20.6**	12.04	pg/dL	8.10–17.10
TSH	**0.008**	1.87	mUI/L	0.3–3.6
PTH	–	16.50	pg/mL	6.50–36.80
C-Peptide	–	1.80	ng/mL	0.85–3.98
Insulin	–	7.0	µU/mL	2.1–22.0
ACTH^c^	**<3**	**4.6**	pg/mL	5–55
Cortisol 8 AM^c^	**5**	**2.1**	µg/dL	9–23
Cortisol 12 AM	–	16.1	µg/dL	9–23
Urinary free cortisol	–	153.0	nmol/24 h	38.0–208.0
GH 1st measurement	–	1.97	ng/mL	0.06–6.88
GH 2nd measurement	–	2.16	ng/mL	0.06–6.88
IGF-1 1st measurement	–	**883.0**	ng/mL	54.0–499.0
IGF-1 2nd measurement	–	**1256.0**	ng/mL	54.0–499.0
PRL 1st measurement	–	250	mUI/L	132–498
PRL 2nd measurement	–	422	mUI/L	132–498
LH	–	5.5	mUI/mL	Follicular phase: 1.9–9.2
FSH	–	8.2	mUI/mL	Follicular phase: 3.5–9.2
17β-Estradiol	–	51.3	pg/mL	Follicular phase: 1–112
Aldosterone	–	5.92	ng/dL	2.52–39.2
PRA	–	**0.40**	ng/mLh	1.5–5.7

Abnormal values are reported in bold

*anti-GAD Ab* anti-glutamic acid decarboxylaseantibodies, *anti-TG Ab* anti-thyroglobulin antibodies, *anti-TPO Ab* anti-thyroid peroxidase antibodies, *Anti-TSHr Ab* anti-TSH receptorsantibodies, *FT3* free triiodothyronine, *FT4* free thyroxine, *TSH* thyroid-stimulating hormone, *PTH* parathyroid hormone, *ACTH* adrenocorticotropic hormone, *GH* growth hormone, *IGF* insulin-like growthfactor, *PRL* prolactin, *LH* Luteinizing hormone, *FSH* follicle-stimulatinghormone, *PRA* plasma renin activity

^a^No hormone replacement therapy initiated

^b^On hydrocortisone (10 mg at 8 AM, 5 mg at 12 PM, 2.5 mg at 6 PM)

^c^Following 1-mg dexamethasone overnight suppression test

**Fig. 2:**
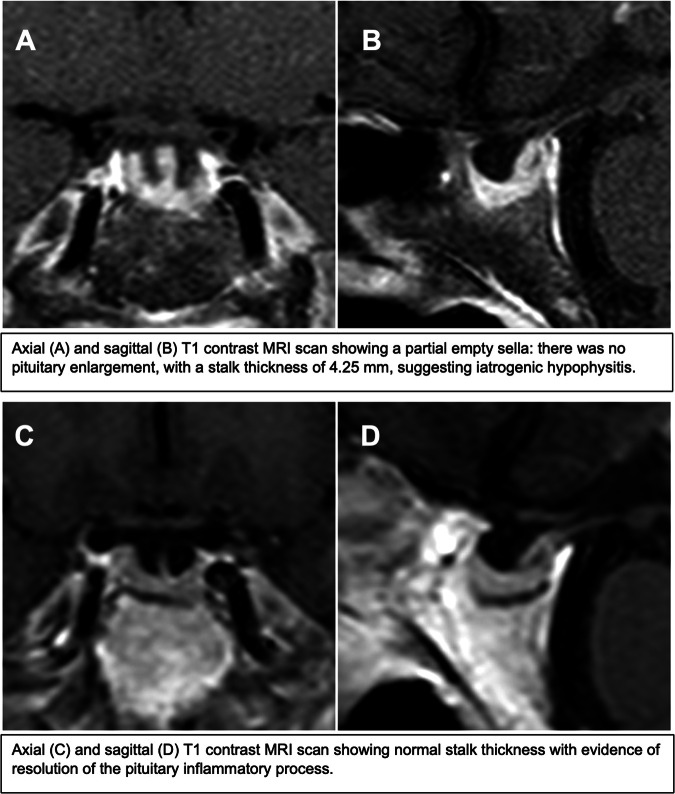
Pituitary MRI. August 2019 (adrenal crisis): panel (**A**, **B**); January 2020: panel (**C**, **D**). MRI magnetic resonance imaging

Magnetic resonance imaging (MRI) of the brain (Fig. 2A, B) revealed no metastatic disease and demonstrated a partial empty sella: there was no pituitary enlargement, with a stalk thickness of 4.25 mm, suggesting iatrogenic hypophysitis. There was no evidence of focal lesions suggesting the presence of pituitary adenoma. Even in the absence of headache, visual disturbance, or mass effect symptoms, ESMO clinical practice guidelines suggest continuing immune checkpoint inhibitors with appropriate hormone replacement therapy [2]; nevertheless, nivolumab was discontinued due to the patient’s decision.

In December 2019, the patient came to our attention, and a complete hormonal screening was performed (Table 1), confirming euthyroidism without levothyroxine replacement therapy, and revealing optimal cortisol replacement and normal PRL levels. Pancreatic endocrine function was preserved, with normal blood glucose and HbA_1c_ levels, which were assessed to exclude iatrogenic diabetes due to nivolumab treatment [3]. Since elevated IGF-1 levels were detected, a 75-g oral glucose tolerance test was performed, which revealed normal GH suppression with GH constantly under 1 ng/mL and nadir under 0.4 ng/mL; the patient had no typical signs or symptoms of acromegaly. At ultrasound evaluation, thyroid nodules were found. The largest thyroid nodule, located at the lower third of the left lobe, was solid, iso-hypoechoic, and measured 14.5_*_15.9_*_17.1 mm and perinodular vascularity; cytological examination revealed a benign result (TIR2). The patient did not have cystic ovaries and showed a regular menstrual cycle.

In January 2020 a new MRI was performed (Fig. 2C, D), with evidence of resolution of the pituitary inflammatory process. ACTH test was then performed, showing only a small increase in serum cortisol levels after cosyntropin (250 µg intramuscular) administration (basal 4.2 µg/dL, peak 8.6 µg/dL) and confirming the need for cortisol replacement therapy. The patient subsequently underwent periodic follow-up at our Endocrinology Outpatient Clinic.

In May 2020, head and neck MRI showed recurrence of the parotid melanoma, and a new surgical intervention was performed with negative resection margins. No further relapses occurred.

In October 2020, the patient underwent thrombolysis for ischaemic stroke and completed a rehabilitation programme, reporting no motor or sensory deficits. The patient was not followed at our department; however, no cause for the ischaemic attack was identified.

In December 2020, she contracted SARS-CoV-2 infection and received the first dose of the ChAdOx1 nCoV-19 COVID-19 vaccine (Vaxzevria, AstraZeneca) on March 5, 2021; this vaccine was later associated with rare episodes of thrombosis with thrombocytopenia syndrome [4].

In April 2021, the patient had another ischaemic stroke and underwent systemic thrombolysis. Echocardiographic examination revealed a left atrial mass that was removed on the following day in the Cardiothoracic surgery unit, with histological finding of myxoma.

Due to the rarity of this neoplasm, in January 2022, a genetic referral was requested to determine the aetiology of the growth of such a variety of tumours in a single patient, specifically suspecting Carney Complex. A heterozygous pathogenic variant [c.491_492del;p.(Val164Aspfs*5)] in exon 5 of the *PRKAR1A* gene was found, confirming the diagnosis. During the genetic referral, due to the autosomal dominant nature of the pathogenic variant, the patient was informed to extend molecular testing to her relatives as well. However, the patient has no children, and, to the best of our knowledge, no relatives have been tested so far.

## Discussion

This case report highlights several unexpected clinical scenarios. First, the patient was initially referred to our clinic due to iatrogenic hypophysitis, resulting in secondary adrenal insufficiency, though she previously suffered from primary hypercortisolism. Hypophysitis is a well-recognized, potentially life-threatening immune-related adverse event (irAE) of immune checkpoint inhibitors, and it appears to be more frequent in patients receiving anti-cytotoxic T-lymphocyte antigen 4 (CTLA-4) monoclonal antibodies or combination therapy with anti-programmed cell-death (PD) protein 1 (PD-1)/anti-PD ligand 1 (PD-L1) plus anti-CTLA-4 immune-checkpoint inhibitors. Recently, immune-related hypophysitis has been increasingly related with anti-PD-1/PD-L1 monoclonal antibodies [5]. As in this case, hypophysitis usually affects anterior or posterior pituitary function permanently, since the production and secretion of pituitary hormones may rarely recover [6]. The typical MRI appearance of hypophysitis can vary, but usually include decreased signal intensity on T1-weighted images, which may indicate infiltration or replacement by inflammatory cells or fibrotic tissue, heterogeneous hyperintensity on T2-weighted images, moderate gland enlargement with symmetrical suprasellar extension, general homogeneous contrast enhancement [7] and thickened, not deviated, pituitary stalk, which is perhaps the strongest predictor of an inflammatory process [8]. The presence of an “empty sella” may be considered as the atrophic outcome following the inflammatory process [9]. As far as it concerns our patient, during the inflammatory phase of hypophysitis the stalk was thicker and there was greater contrast enhancement than after the resolution of the inflammatory process, in which the picture of “empty sella” prevailed. The stalk was substantially in axis in both evaluations, and there was no evidence of focal lesions suggesting the presence of pituitary adenoma. In our patient, autoimmunity assessment was negative in line with T cell-mediated cytotoxicity due to use of the immune checkpoint inhibitor. Indeed, endocrinopathies and consequent hormonal deficiencies are generally accepted as a trade-off for increased survival in patients treated with immunotherapy for metastatic melanoma, and clinicians should be aware of these irAEs. Accumulating clinical evidence is driving the formulation of more and more refined guidelines and recommendations to identify potential predisposing factors, ideal monitoring, and adequate management of irAEs due to checkpoint inhibitors [2, 10–15].

Furthermore, our patient had high IGF-1 levels, with a normal suppression of GH during OGTT and neither clinical signs of symptoms of acromegaly, nor MRI findings of pituitary adenoma. In up to 70% of patients with CNC, subtle abnormalities of the GH axis, including elevated (asymptomatic) baseline GH or IGF-1 or non-suppressible GH to an OGTT can be observed [16]. This hormonal imbalance is related to the occurrence of a certain degree of pituitary hyperplasia, which may be present before tumour development [16, 17]. Acromegaly caused by a GH-secreting pituitary adenoma is a relatively uncommon presentation of CNC, occurring in less than 10% of individuals with this condition [18, 19]. In a case series of 49 patients with acromegaly due to CNC [20], there was a roughly equivalent ratio of macroadenomas (n = 27) and microadenomas (n = 24), whereas non-CNC acromegaly typically shows a higher prevalence of macroadenomas (ranging from 70 to 90% depending on the study) [21].

Moreover, patients with CNC do not appear to have predisposition to skin cancers, whereas this is not the case with other genetic syndromes associated with melanotic and other cutaneous lesions. Our patient was diagnosed with parotid malignant melanoma, which is not a typical neoplasm of CNC in contrast to melanotic schwannoma; these two different neoplasms may be confused with each other due to similar histopathology [22]. The verification should be performed by a pathologist experienced in Carney complex, and staining for *PRKAR1A* protein may be helpful to distinguish between these two types of tumours [23]. In our case, histopathological diagnosis of melanoma was confirmed twice by two pathologists from different hospitals. However, being well aware that melanotic schwannoma can be confused with malignant melanoma, we recommended histopathology verification, but the patient refused our proposal.

In addition, the patient had encapsulated intracystic papillary breast carcinoma with rare foci of infiltration of surrounding stroma, and no *BRCA1/2* mutation. At histological examination breast myxomatosis was not mentioned by the pathologist. Breast myxomatosis is the most common mammary tumour in CNC, occurring in ~20% of female patients after puberty, and is often bilateral [24]. Ductal adenoma may also occurs, with a frequency of ~3% in female patients [25]. Both are considered benign lesions. Few reports mention malignant breast cancer in CNC female patients [26, 27].

CNC should be suspected in individuals with two or more major diagnostic criteria [28], which include spotty skin pigmentation with typical distribution (lips, conjunctiva and inner or outer canthi, vaginal and penile mucosa), myxoma, cardiac myxoma, breast myxomatosis or fat-suppressed MRI findings suggestive of this diagnosis, PPNAD, acromegaly as a result of growth hormone (GH)-producing adenoma, large-cell calcifying Sertoli cell tumour (LCCSCT) or characteristic calcification on testicular ultrasound, thyroid carcinoma or multiple, hypoechoic nodules on thyroid ultrasound in a child younger than age 18 years, psammomatous melanotic schwannomas (PMS), blue naevus, epithelioid blue naevus, breast ductal adenoma and osteochondromyxoma. In the case of our patient, due to confounding clinical history details (such as adrenocortical adenoma vs PPNAD and parotid melanoma vs PMS) and the primary reason for her visit (iatrogenic hypophysitis), the diagnosis was made only after the finding of atrial myxoma, a rare disease which is often found in CNC. Primary cardiac tumours are extremely rare (0.0017%–0.19% of autopsied cases), and 75% of these are benign. Cardiac myxomas account for approximately 50% of primary benign cardiac tumours and are often solitary, with the left atrium being the most common site of origin [29]. Therefore, the detection of the atrial myxoma by the Cardiothoracic surgery unit was crucial to provide the final clue to make the diagnosis of CNC.

The patient’s *PRKAR1A* gene mutation has been previously described and is associated with a highly variable clinical spectrum. Some Authors reported absence of alterations typical of the CNC [30], while others described cases of CNC complicated with PPNAD without Cushing’s syndrome [31], and cases of atrial myxomas, either extensive [32] or recurrent [33].

In conclusion, in this case, the coordinated efforts of a multidisciplinary team led to the diagnosis of a rare disease with a wide spectrum of life-threatening clinical manifestations and unexpected clinical scenarios.
